# Extreme Divergence of *Wolbachia* Tropism for the Stem-Cell-Niche in the *Drosophila* Testis

**DOI:** 10.1371/journal.ppat.1004577

**Published:** 2014-12-18

**Authors:** Michelle E. Toomey, Horacio M. Frydman

**Affiliations:** Department of Biology, Boston University, Boston, Massachusetts, United States of America; The Pennsylvania State University, United States of America

## Abstract

Microbial tropism, the infection of specific cells and tissues by a microorganism, is a fundamental aspect of host-microbe interactions. The intracellular bacteria *Wolbachia* have a peculiar tropism for the stem cell niches in the *Drosophila* ovary, the microenvironments that support the cells producing the eggs. The molecular underpinnings of *Wolbachia* stem cell niche tropism are unknown. We have previously shown that the patterns of tropism in the ovary show a high degree of conservation across the *Wolbachia* lineage, with closely related *Wolbachia* strains usually displaying the same pattern of stem cell niche tropism. It has also been shown that tropism to these structures in the ovary facilitates both vertical and horizontal transmission, providing a strong selective pressure towards evolutionary conservation of tropism. Here we show great disparity in the evolutionary conservation and underlying mechanisms of stem cell niche tropism between male and female gonads. In contrast to females, niche tropism in the male testis is not pervasive, present in only 45% of niches analyzed. The patterns of niche tropism in the testis are not evolutionarily maintained across the *Wolbachia* lineage, unlike what was shown in the females. Furthermore, hub tropism does not correlate with cytoplasmic incompatibility, a *Wolbachia*-driven phenotype imprinted during spermatogenesis. Towards identifying the molecular mechanism of hub tropism, we performed hybrid analyses of *Wolbachia* strains in non-native hosts. These results indicate that both *Wolbachia* and host derived factors play a role in the targeting of the stem cell niche in the testis. Surprisingly, even closely related *Wolbachia* strains in *Drosophila melanogaster*, derived from a single ancestor only 8,000 years ago, have significantly different tropisms to the hub, highlighting that stem cell niche tropism is rapidly diverging in males. These findings provide a powerful system to investigate the mechanisms and evolution of microbial tissue tropism.

## Author Summary

Microbes evolve to infect structures favoring their transmission in host populations. A large fraction of insects are infected with *Wolbachia* bacteria. Usually *Wolbachia* are transmitted the same way we inherit our mitochondria, via the eggs from the mother. In fruit flies, to favor maternal transmission, *Wolbachia* infect the microenvironment containing the egg producing stem cells, called the “stem cell niche”. Targeting of the stem cell niche is evolutionary conserved in female fruit flies, observed in all *Wolbachia* strains analyzed to date. Remarkably, in males, we find many *Wolbachia* strains not infecting the stem cell niche present in the testis. We report a surprising diversity in stem cell niche infection in males, contrasting with extreme conservation in females. We further show that even closely related *Wolbachia* strains in *D. melanogaster* display rapidly evolving patterns of stem cell niche targeting in males. Understanding the molecular mechanisms driving these differences will identify sex specific features of stem cell niche biology. Because *Wolbachia* promote insect resistance against human diseases transmitted by mosquitos, *Wolbachia* are becoming a valuable tool in the control of several diseases, including Dengue and malaria. Knowledge emerging from this research will also provide novel tools towards *Wolbachia* based strategies of disease control.

## Introduction

The evolutionary interests of males and females are frequently divergent. Sexual conflict arises when phenotypes that enhance the reproductive success of one sex reduces the fitness of the other sex [1]. A well-characterized example in *Drosophila* is sperm competition between males. Sperm competition results in rapid evolution of sperm proteins which up-regulate females' egg-laying rate and reduces her desire to re-mate with another male. However, these proteins also shorten the female's lifespan reducing her fitness [reviewed by 2].

Vertically transmitted reproductive parasites, such as *Wolbachia*, can also cause sexually divergent phenotypes in males and females. *Wolbachia* are obligate intracellular bacteria present in a large fraction of insects, as well as spiders, mites, crustaceans, and filarial worms. They are primarily vertically transmitted from mother to offspring in a manner analogous to mitochondrial inheritance, although there is extensive evidence of horizontal transmission in nature [3], [4]. For intracellular bacteria, vertical transmission often favors infected female hosts, which is also the case for *Wolbachia* [5]. There are several *Wolbachia*-induced phenotypes favoring the infected female, including parthenogenesis, feminization, male killing, and cytoplasmic incompatibility [6]. Each of these phenotypes ultimately results in the spread of more infected female hosts. In such cases, maternally transmitted bacteria can act as selfish genetic elements driving sexual conflict [5].

For successful vertical transmission, *Wolbachia* need to be present in the eggs laid by infected females. It has been shown in *Drosophila* that *Wolbachia* display a strong tropism for the germline, in particular, the oocyte, to ensure a high percentage of vertical transmission [7]–[10]. Although vertical transmission is prevalent, *Wolbachia* also can spread horizontally across individuals and species [3], [11], [12]. Colonization of the germline is a prerequisite for the infection to become successfully established into a population. We have previously shown that upon recent infection, *Wolbachia* colonize the stem cell niches in the *Drosophila* ovary, favoring vertical transmission after horizontal transfer [13]. Furthermore, stem cell niche tropism in the ovary is a highly evolutionarily conserved phenotype across the *Drosophila* genus, present in 100% of ovaries analyzed [14]. *Wolbachia* also infect the putative stem cell niches in the ovaries of other species, such as the bedbug and leafhopper [15], [16] indicating that the selective pressure for *Wolbachia* targeting of ovarian stem cell niches to favor transmission extends beyond the *Drosophila* genus.

*Wolbachia* have also been shown to display tropism to the stem cell niche present in the testis in *Drosophila mauritiana* [17]. However, the conservation of this phenotype across the *Drosophila* genus is unknown. Here we show that the evolutionary conservation of stem cell niche tropism present in females is not maintained in the male lineage. In fact, *Wolbachia* niche tropism in the testis, compared to the female results, represents a pronounced sexual dimorphism in the evolutionary history of *Wolbachia* stem cell niche tropism. Furthermore, we′ve identified that both *Wolbachia* and host factors modulate hub tropism in this system. Finally, we show that very closely related *Wolbachia* strains infecting the same host differ significantly in the densities at which they colonize the hub, indicating that hub tropism is a rapidly diverging phenotype in males.

## Results

### *Wolbachia* targeting of the hub in the *Drosophila* testis is not pervasive

**Figure 1:**
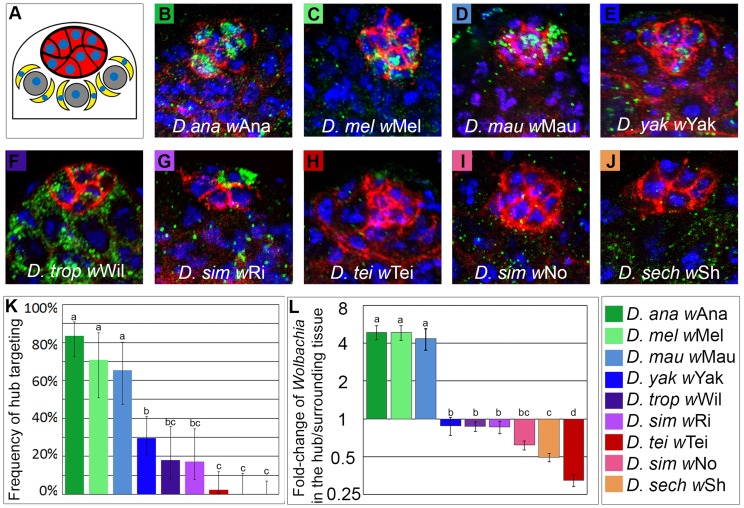
Diverse *Wolbachia* strains infect the hub of various *Drosophila* species at different frequencies and densities. (A) Diagram of the testis apical tip, with cell nuclei in blue. The germline stem cells (GSCs, grey) and cyst stem cells (CySCs, yellow) reside at the hub (red). (B-J) Representative images of *Wolbachia* (green) hub tropism in 9 *Drosophila* species (hub marker, red; DNA, blue). (K) Quantification of the frequency of *Wolbachia* hub tropism in each *Drosophila* species (Error bars represent 95% confidence intervals). Letters indicate statistically significant groups (two-sample test for proportions). (L) Quantification of *Wolbachia* density in the hub, normalized to the surrounding tissue (Error bars represent SEM for average density across all samples). Letters indicate statistically significant groups (pairwise T-tests). [For each host/*Wolbachia* pair, abbreviations are as follows: *D. ana w*Ana, *Drosophila ananassae* infected with *Wolbachia ananassae*. See S1 Table for details.]

In the testis, the germline stem cells (GSCs) and cyst stem cells (CySCs) reside at the “hub”, a structure at the apical tip of the testis (Fig. 1A). The hub is a group of 10 to 16 somatically derived cells forming the microenvironment supporting the stem cells, referred to as the niche [18]. It has been shown that the GSCs receive maintenance signals from both the hub and the CySCs, hence both are considered to be part of the stem cell niche for the GSCs. However for the context of this study, niche tropism in the testis refers to *Wolbachia* infection of the hub only. To investigate whether *Wolbachia* niche tropism is as pervasive in the hub, as previously shown in the ovary [14], we surveyed various *Drosophila* species infected with different strains of *Wolbachia* (Fig. 1; S1 Dataset; see S1 Table for the sources for the stocks used in this analysis).

Using confocal imaging and immunohistochemistry, we analyzed the density of *Wolbachia* infection in the hub cells as compared to the density of *Wolbachia* in the surrounding tissue (see Materials and Methods). We found that *Wolbachia* target the hub at varying frequencies and densities across the *Drosophila* genus (Fig. 1, S2 Table, S1 Dataset). 3 out of 9 species showed very little to no *Wolbachia* infection in the hub (Fig. 1 H–J, quantification in K), indicating that hub tropism is not pervasive across the *Drosophila* genus. 6 out of 9 species analyzed, however, did have *Wolbachia* tropism to the hub, ranging from 17% of niches infected to 95% of niches infected (Fig. 1 B–G, K, see also Materials and Methods). The 6 *Drosophila* species- *Wolbachia* strain pairs with hub tropism fall into two groups with significantly different frequencies and densities of tropism: 3 had very high frequencies and densities of hub infection: *D. ananassae w*Ana, *D. melanogaster w*Mel, and *D. mauritiana w*Mau; and 3 had moderate frequencies of *Wolbachia* tropism to the hub: *D. yakuba, w*Yak, *D. tropicalis w*Wil, and *D. simulans w*Ri. In the ovary, tropism to the somatic stem cell niche is found at high frequencies in every individual of all *Drosophila* species analyzed [14]. In contrast, tropism for the hub is found in only a fraction of the species analyzed.

### Hub targeting does not correlate with germline stem cell niche tropism in the ovary

**Figure 2:**
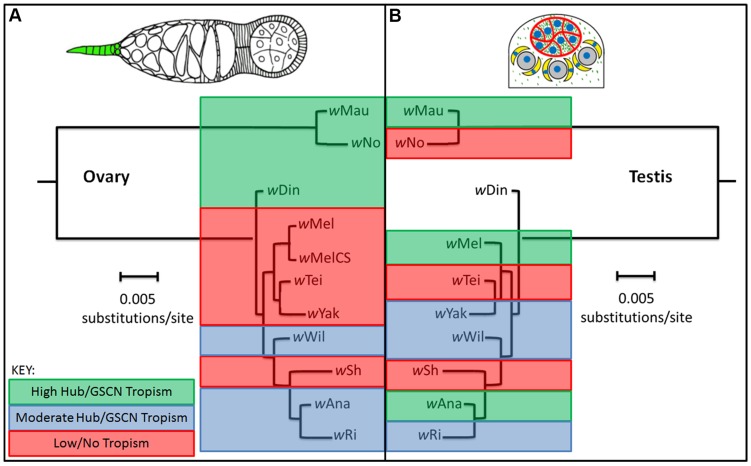
Comparison of evolutionary conservation of niche tropism in males and females. Diagrams of ovary and testis displaying *Wolbachia* tropism (green) to the GSCN and hub, respectively, are shown at the top. Ovary data adapted from [14]. Color key at top right: Green  =  High hub/GSCN tropism, Blue  =  moderate hub/GSCN tropism, Red  =  low/no hub/GSCN tropism (see S3 Table for details). Pattern of *Wolbachia* tropism is evolutionarily conserved in the female ovaries (A), but not in the testis (B). There is no clear correlation of tropism pattern with the *Wolbachia* phylogeny in the testis as was seen in the ovaries (P = 0.773).

Similar to the results for hub tropism, the frequency of tropism to the germline stem cell niche (GSCN) in the ovary was shown to be variable across the *Drosophila* genus (Fig. 2A and [14]). We reasoned that *Wolbachia* tropism to the hub in the testis could simply be a byproduct of GSCN targeting in the ovaries. Interestingly, however, the presence of hub tropism does not correlate with the presence GSCN tropism (S3 Table, Correlation Test, p = 0.773). Although tropism in males and females is correlated in some strains (5 out of 9, e.g. *w*Mau displays high frequencies of both hub tropism and GSCN tropism and *w*Sh does not have tropism to either the hub or the GSCN), there are also others that do not (4 out of 9). The *Wolbachia* strain displaying one of the highest frequencies of GSCN tropism in the ovary (*w*No, 99% [14]), displays no tropism to the hub (0%, Fig. 1 I and K). Conversely, a *Wolbachia* strain displaying a high frequency of tropism to the hub (*w*Mel, 71%, Fig. 1 C and K) does not target the GSCN in the ovary (1%, [14]). These data reveal that *Wolbachia* stem cell niche tropism does not correlate with GSCN tropism in the female.

### Hub tropism phenotype is independent of host and bacterial phylogenies

Previously, we have shown that the pattern of GSCN tropism is evolutionarily conserved across the *Wolbachia* lineage ([14] and Fig. 2). To assess whether hub tropism was also conserved across the *Wolbachia* lineage, we aligned the frequencies of hub tropism on the *Wolbachia* phylogenetic tree (Fig. 2). We quantified the correlation of hub tropism pattern with the *Wolbachia* phylogeny using a computer simulated model of randomized character distributions to compare with the distribution of niche tropism pattern on each of the phylogenies, as previously described [14]. We found that it is highly probable that the distribution of hub tropism is completely independent of the *Wolbachia* phylogeny (S2 Fig.). Similarly, when we compared hub tropism to the *Drosophila* phylogeny, we found no clear correlation between the two (S3 Fig.). Quantification of the relationship revealed that frequency of hub tropism bears no correlation with the *Drosophila* phylogeny (S4 Fig.).

### Hub tropism does not correlate with cytoplasmic incompatibility

An important *Wolbachia* related phenotype that also bears no correlation with host or microbial phylogenies is cytoplasmic incompatibility (CI). CI is a reproductive phenotype resulting in reduced embryo hatching when a *Wolbachia* infected male mates with an uninfected female. We examined the possibility of a correlation between tropism to the hub and CI by comparing our tropism data to previously published reports on the levels of CI across the *Drosophila* genus (S4 Table) [19]–[23]. This analysis shows that some species with high levels of CI have different levels of tropism (i.e. *w*Sh and *w*Ri have 0% and 17% hub tropism, respectively). Conversely, some species with low levels of CI also have a wide range of hub tropism phenotypes (i.e. *w*Tei and *w*Mau have 2.3% and 71% hub tropism frequencies, respectively). Although hub tropism is highly divergent even amongst closely related strains of *Wolbachia*, similar to CI, there does not seem to be a correlation between these two phenotypes (S4 Table, Correlation test, p = 0.267).

### Both host and bacterial factors can influence hub tropism

**Figure 3:**
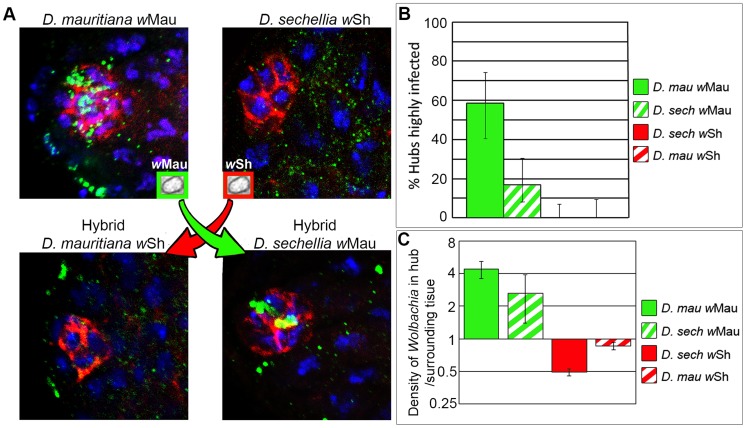
Both host and *Wolbachia* factors influence hub tropism. (A) Representative images of *Wolbachia* tropism to the hub in parental *D. mauritiana* and *D. sechellia* testis (top row) and F_5_ hybrid testis (bottom row) [*Wolbachia*, green; hub marker, red; DNA, blue]. Red and green arrows represent direction of *Wolbachia* transfer. (B) Quantification of frequency of hub tropism. Solid and hatched bars represent the parental and hybrid host species, respectively. Error bars represent 95% confidence intervals. Fisher Exact tests indicate that both the host genetic background and the *Wolbachia* strain have a significant effect on hub tropism (p = 8.309×10^−5^ and p = 2.267×10^−10^, respectively). (C) Quantification of *Wolbachia* density in the hub, normalized to the surrounding germline and soma. Linear regression analysis indicates that the *Wolbachia* strain, rather than the host genetic background, modulates *Wolbachia* density in the hub (P = 0.045 and P = 0.56, respectively).

We next aimed to elucidate if host or bacterial factors influence the highly dynamic nature of the hub tropism phenotype. To investigate this question, *Wolbachia* strains backcrossed into a different host were used to assess *Wolbachia* strain versus host background influence on hub tropism, as previously described [14]. *D. mauritiana w*Mau, which displays hub tropism (Fig. 1D and Fig. 3) and *D. sechellia w*Sh, which does not display hub tropism (Fig. 1J and Fig. 3) and their hybrid offspring were utilized in this study (See Material and Methods).

*Wolbachia* strain *w*Sh, infecting its native host, *D. sechellia*, and its non-native host, *D. mauritiana*, displays no hub localization, regardless of host genetic background (Fig. 3, S5 Table). This result suggests that *Wolbachia w*Sh is incapable of hub tropism in either species. However it does not rule out the possibility that the hosts share a mechanism for excluding *w*Sh from the hub. Therefore, a lack of tropism in both hosts cannot provide insight into whether the host or microbe is providing factors contributing to hub tropism.

The analysis of *w*Mau hub tropism allows further probing into this question. *Wolbachia* strain *w*Mau infecting its native host, *D. mauritiana*, and its non-native host, *D. sechellia*, displays tropism for the hub, suggesting that the *Wolbachia* strain is driving this phenotype. However, the frequency of targeting in the hybrid host is 3-fold lower than in the native host (Fig. 3C, green bars). Statistical analysis of frequency data indicates that both host genetic background and *Wolbachia* strain can significantly affect the frequency of hub tropism (Fisher's exact test, p = 8.309×10^−5^ and p = 2.267×10^−10^, respectively). These results are in contrast to previous data in the ovaries where only the *Wolbachia* strain drives tropism. *w*Mau can efficiently target the GSCN in the ovary of both its native and hybrid host, greater than 80% of niches infected, regardless of the host genetic background [14]. The *w*Mau frequency data in the male support the hypothesis that the *Wolbachia* strain is directing hub tropism. However, because the frequency of targeting is not as robust in the hybrid host compared to its native host, a role for the host is also implicated.

In relation to *Wolbachia* density in the hub, the data indicate that the *Wolbachia* encoded factors play a major role in both native and hybrid hosts. The overall density at which *w*Mau infect the hub is conserved (Fig. 3 B and C, native host solid green bar, hybrid host hatched green bar, S4 Table). Similarly, *w*Sh hub titers, compared to the surrounding tissue, are less than 1 in both native and hybrid hosts (Fig. 3 B and C, native host solid red bar and hybrid host hatched red bar, S4 Table). Linear regression analysis of density data indicates that the *Wolbachia* strain, rather than the host genetic background, modulates *Wolbachia* density in the hub (P = 0.045 and P = 0.56, respectively). With respect to both frequency and density, the overall data reveal that factors encoded by both the host species and the *Wolbachia* strain influence hub tropism in the *Drosophila* testis.

### *Wolbachia* strain specific factors are sufficient for differences in hub tropism

**Figure 4:**
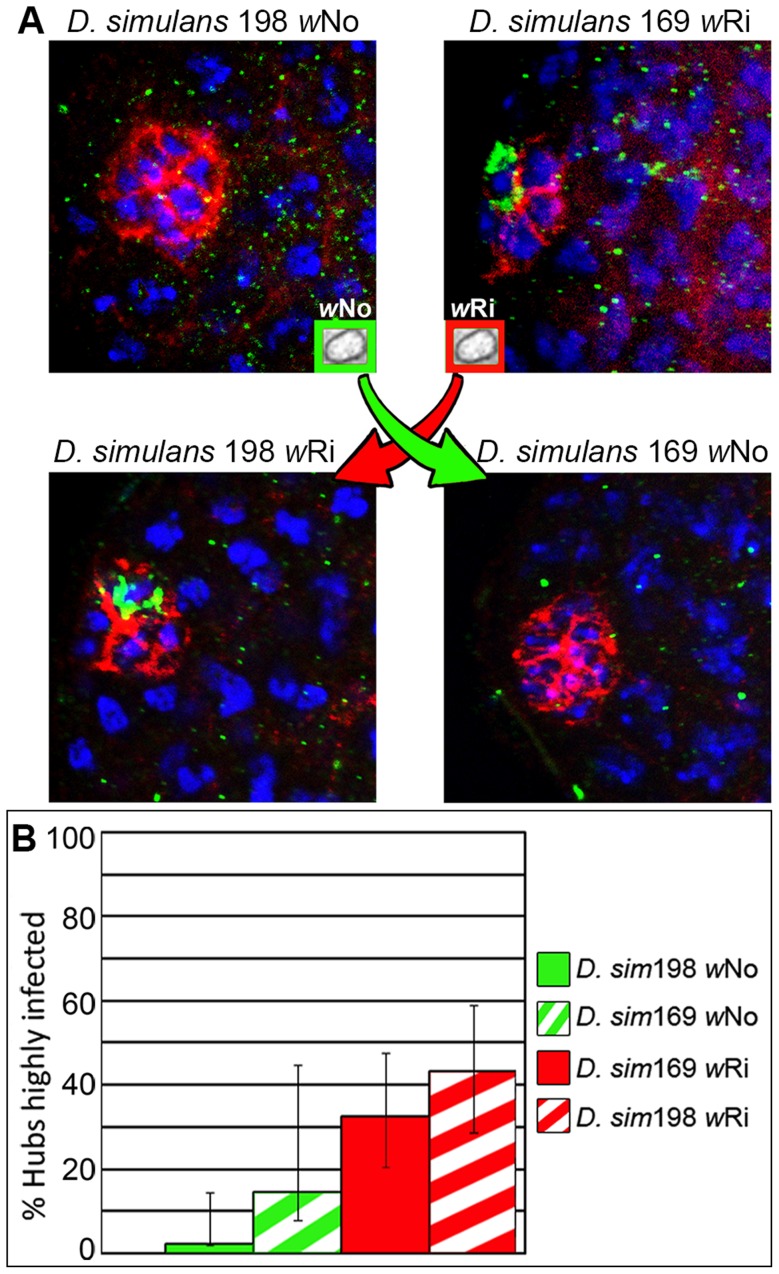
*Wolbachia* strain directs niche targeting in closely related *Drosophila* strains. (A) Representative images of *Wolbachia* tropism to the hub in parental *D. simulans* strains 198 and 169 testis (top row) and F_5_ hybrid testis (bottom row) [*Wolbachia*, green; hub marker, red; DNA, blue]. Red and green arrows represent direction of *Wolbachia* transfer. (B) Quantification of frequency of hub tropism. Solid and hatched bars represent the parental and hybrid host species, respectively. Error bars represent 95% confidence intervals. Logistical regression confirms *Wolbachia* factors have a significant effect on hub tropism as compared to the host genetic background (p = 0.0000552 and p = 0.927 respectively).

To further investigate the role of *Wolbachia* on hub tropism, we then analyzed different *Wolbachia* strains in the same host species. We took advantage of *D. simulans,* which is a host to many different *Wolbachia* strains. We investigated two strains of *D. simulans* flies differentially infected with *w*Ri and *w*No and their backcrossed offspring. Flies were backcrossed to account for any genomic divergence between host strains, as previously described [14]. *D. simulans* flies infected with *Wolbachia w*Ri display hub tropism in about 33% and 43% of hubs analyzed for the parental and backcrossed hosts, respectively (Fig. 4, S6 Table). *D. simulans w*No displays hub tropism infrequently (2% and 15% of hubs highly infected for the parental and backcrossed hosts, respectively, Fig. 4, S6 Table). Although the frequencies of hub tropism for each *Wolbachia* strain increase in the backcrossed hosts, the general trend remains, where *w*Ri targets the hub at a higher frequency than *w*No. To quantify the relative contributions of host and bacterial factors towards hub tropism, logistical regression was performed. *Wolbachia* factors have a significant effect on hub tropism as compared to no significance of the host genetic background in the *D. simulans* hybrid flies (p = 0.0000552 and p = 0.927 respectively). These results indicate that when host factors are kept constant, *Wolbachia* strain factors are sufficient to significantly modulate the frequency of hub tropism.

### Hub tropism is a rapidly diverging phenotype

In the previous analyses of hybrid crosses, hub tropism of distantly related *Wolbachia* strains were compared, first with different host species (Fig. 3), then within the same host species (Fig. 4). These results indicate that although the fly host can play a role in hub tropism, *Wolbachia* can significantly affect tropism on its own. In both cases, we were comparing *Wolbachia* strains from the A and B supergroups. We next investigated if the observed diversity of niche tropism is still present between more closely related *Wolbachia* strains. To address this question, we analyzed hub tropism of several *Wolbachia* strain variants infecting *Drosophila melanogaster* that diverged from a single ancestor within the last 8,000 years [24], [25].

**Figure 5:**
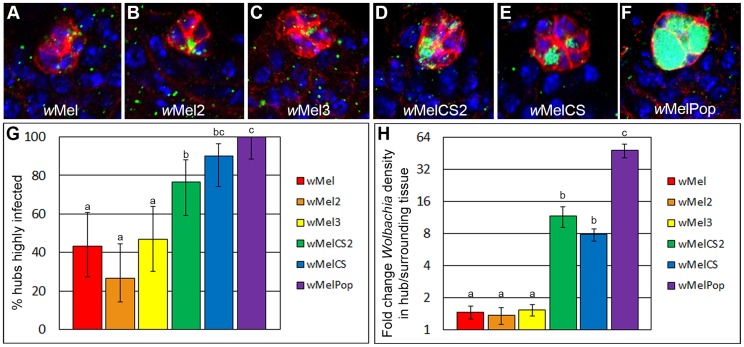
Closely related *Wolbachia* strains display rapidly divergent hub tropism phenotypes in *Drosophila melanogaster*. (A-F) Representative images of *w*Mel-like strains: *w*Mel, *w*Mel2, *w*Mel3; and *w*MelCS-like strains: *w*MelCS2, *w*MelCS and *w*MelPop infecting *D. melanogaster* hubs [*Wolbachia*, green; hub marker, red; DNA, blue]. (G) Quantification of frequency of hub tropism. The three *w*Mel-like *Wolbachia* strains target the hub at similar frequencies, significantly different from the *w*MelCS-like strains. Frequencies with different letters are significantly different (two-sample test for proportions, error bars represent 95% confidence intervals). (H) Quantification of density of *Wolbachia* infecting the hub. The three *w*Mel-like *Wolbachia* strains target the hub at similar densities, and are significantly different from the *w*MelCS-like strains. Means with different letters are significantly different from one another as determined by a t-test, error bars represent SEM.

Hub tropism of *w*Mel-like (*w*Mel, *w*Mel2, and *w*Mel3) and *w*MelCS-like (*w*MelCS, *w*MelCS2, and *w*MelPop) *Wolbachia* strains were analyzed. These *Wolbachia* strains were introgressed into the same *D. melanogaster* genetic background with the same microbiota [25]. The data reveal that the three *w*Mel-like *Wolbachia* strains have significantly different tropism phenotypes from the *w*MelCS-like strains (Fig. 5, S7 Table). The *w*Mel-like strains target the hub at similar frequencies, between 25% and 50%, and at similar densities, about 1.5-fold higher than the surrounding tissue. The *w*MelCS-like strains target the hub at significantly higher frequencies (P<0.05) and densities (P<0.001) than the *w*Mel-like strains. Within the *w*MelCS-like group, *w*MelPop targets the hub at a significantly higher frequency (100%) than *w*MelCS2 (77%; P = 0.005), but not *w*MelCS (90%). However, *w*MelPop targets at a significantly higher density than both *w*MelCS and *w*MelCS2 (P<0.0001; S1 Movie). Interestingly, *w*MelPop densities increase to the point where the hub cells burst open in approximately 20% of hubs (S5 Fig. and S2 Movie). The finding that the *w*Mel-like and *w*MelCS-like *Wolbachia* variants, all derived from a single ancestor only 8,000 years ago, have significantly different frequencies and densities of targeting indicates that hub tropism is a rapidly diverging phenotype.

## Discussion

A fundamental aspect of *Wolbachia*-host interactions is the type of tissue preferentially infected by the bacteria. We have previously shown that *Wolbachia* tropism to the stem cell niches in the female *Drosophila* ovaries is important for vertical transmission, and that this tropism is ubiquitous across the *Drosophila* genus. Furthermore, closely related *Wolbachia* strains tend to display the same patterns of tropism in the ovary, indicating the importance of maintaining this phenotype for vertical transmission [14].

If the major role of niche tropism is related to *Wolbachia* transmission, evolutionary theory predicts that there should be reduced selective pressure to maintain niche tropism in males, since *Wolbachia* is not transmitted through the sperm. Patterns of *Wolbachia* niche tropism in the filarial nematode (*B. malayi*, *D. immitis*, *L. sigmondontis, M. unguiculatus*, and *O. dewittei japonica*) support this concept, where *Wolbachia* colonization of the distal tip cell (the nematode equivalent of the stem cell niche) and subsequent germline invasion occurs only in females [26]. In agreement, the results shown here indicate a reduced level of conservation of hub tropism phenotype, contrasting with previous observation in females [14]. The stem cell niches in the ovary and testis are well characterized and have several signaling pathways in common [27]. The robust sexual dimorphism in the evolutionary conservation of niche tropism, indicates that *Wolbachia* could be recognizing novel sex specific differences in these cells [28].

*Wolbachia*-induced host phenotypes related to stem cell biology and testis physiology have been previously described [17], [23]. We investigated whether hub tropism correlates with those known *Wolbachia-*related reproductive phenotypes. Because GSCN tropism in the ovary was shown to not be ubiquitous across the *Drosophila* genus, we reasoned that hub tropism could simply be a byproduct of GSCN tropism in the female. However, the frequencies of GSCN and hub tropism only correlate in 5 out of the 10 species and are not statistically significant.

On the cellular level, another phenotype we have previously shown was a *Wolbachia-*dependent increase in the rate of germline stem cell division (GSCD) in the ovaries of *D. mauritiana.* Although a similar trend exists in the *D. mauritiana* testis, the up-regulation of GSCD was not shown to be significant, showing a lack of conservation of a phenotype derived in the females to boost their spread [17].

A third important *Wolbachia* mediated phenotype, cytoplasmic incompatibility (CI), is a consequence of *Wolbachia* modification of sperm during spermatogenesis, causing embryonic lethality of uninfected eggs fertilized by sperm from infected males [reviewed by 29]. Although the precise mechanism is not well understood, the sperm from infected males is modified (mod^+^) and an infected egg with the appropriate rescue factor (resc^+^) is required for embryo viability [30], [31]. Several lines of evidence suggest that the modification of the sperm occurs at the chromatin level [32]–[34]. Extensive analyses of *Wolbachia* population dynamics and localization during spermatogenesis have demonstrated that CI is a non-cell autonomous effect caused by a diffusible *Wolbachia* factor during spermatogenesis [35]. Interestingly, local factors secreted by the hub can act on the germline stem cell. Since niche factors are extrinsic to the stem cell, they can affect the testis germline stem cell and consequently their sperm-forming progeny in a non-cell autonomous fashion. Niche factors have also been shown to cooperate with chromatin remodeling complexes towards control of germline stem cell maintenance and differentiation [36]. Therefore, we attempted to correlate our tropism data with published data regarding CI levels of several *Wolbachia* strains across the *Drosophila* genus. However, we found no correlation between *Wolbachia* hub tropism and CI, suggesting that *Wolbachia*'s presence in the hub is not required for the CI effect. This suggests that either *Wolbachia* factors modify the sperm later in spermatogenesis or if *Wolbachia*-derived factors are affecting early spermatogenesis events towards CI, it is independent of *Wolbachia* infection of the niche.

Literature shows that both the host species and *Wolbachia* strains have rapidly evolving aspects that could contribute to the dynamic evolutionary changes in *Wolbachia* hub targeting shown here. Regarding the host, several testis specific genes, male seminal fluid proteins, and spermatogenesis genes have been shown to be rapidly evolving [37]. Furthermore, proteins related to GSC biology are also undergoing recurrent positive selection [38]. From the perspective of the bacteria, *Wolbachia* genomic analyses suggest that these bacteria have one of the most highly recombining intracellular bacterial genomes, with many genomic differences between closely related strains [39]–[42].

We investigated the relative contribution of both host and bacterial factors towards hub tropism phenotype. Unlike in the ovary where host derived factors did not play a role [14], in the testis, host factors could not be ruled out. When comparing distantly related *Wolbachia* strains and host species (*D. mauritiana* and *D. sechellia* hybrid lines), the data indicate that both host and *Wolbachia* derived factors contribute to the differences in hub tropism. One possibility is that there is selective pressure on the host driving rapid evolution of the hub intracellular environment to counteract negative effects of *Wolbachia* colonization of the testis niche. Although there is no evidence in the literature for positive selection of hub proteins, genes in the neighboring germline stem cell have been shown to be undergoing positive selection [38], [43]. Independent of differential host factors, we were able to confirm *Wolbachia's* role in hub tropism. By comparing distantly related *Wolbachia* strains in the same host species (*D. simulans* lines), we were able to confirm that *Wolbachia* derived factors significantly modulate hub tropism.

To assess how quickly this modulation of hub tropism can evolve, we investigated if very closely related *Wolbachia* strains that have recently diverged could display diverse hub tropism phenotypes. Several variants of the *w*Mel strain of *Wolbachia* naturally infecting *D. melanogaster* exist [44], [45]. Due to strict maternal transmission, congruent *Wolbachia* and mitochondrial lineages made it possible to trace these lineages back to a single common *D. melanogaster* ancestor existing around 8,000 years ago [24], [25]. We investigated hub tropism of *w*Mel-like (*w*Mel, *w*Mel2, and *w*Mel3) and *w*MelCS-like (*w*MelCS, *w*MelCS2 and *w*MelPop) *Wolbachia* strains which have been shown to induce differential protection against viruses [25]. The *w*Mel-like and *w*MelCS-like subgroups can be separated into three statistically distinct groups based on their density of hub infection (1: *w*Mel, *w*Mel2, and *w*Mel3; 2: *w*MelCS and *w*MelCS2; 3: *w*MelPop), indicating that they have evolved distinct cellular tropisms. These data demonstrate that hub tropism is a rapidly diverging phenotype.

The fast paced changes in the hub tropism phenotype during the evolution of these different *Wolbachia* strains raises the questions of what mechanisms are driving these rapid changes and is adaptive evolution occurring. If *Wolbachia* tropism for the hub is causing an unfavorable phenotype in the host, a molecular arms race will result where both the host and microbe will rapidly evolve [46], [47]. We did not find any correlation of hub tropism with CI, germline stem cell division, or with other obvious testis related phenotypes. It is possible that hub tropism may have a phenotypic effect on the host, but at the moment these are unknown and we have no evidence supporting adaptive evolution in response to a host-microbe arms race driving rapid changes in hub tropism in *w*Mel strains.

Another possibility is that genetic drift is driving the extreme divergence in hub tropism that we report here. At every generation, from embryonic development through the mature egg, *Wolbachia* undergoes several bottlenecks: only the *Wolbachia* present in the germplasm of the embryo will colonize the primordial germ cells [8], [10]. Within the germline, only the *Wolbachia* present in the oocyte is transmitted to the progeny [7], [9], [10]. This effectively reduces the genetic effective population sizes and increases the rate of fixation of mutations by drift. There are several studies highlighting the role of genetic drift driving high rates of genome sequence evolution in vertically transmitted endosymbionts [reviewed by 48]. The data presented here suggest that mutations that are neutral regarding niche targeting in the female may affect niche tropism in the male. If these mutations do not affect *Wolbachia* overall fitness in the females and do not interfere with transmission, they can be fixed by drift and result in significant niche tropism evolution in males.

At the moment it is difficult to identify the specific molecular underpinnings resulting in the differences in niche tropism phenotypes between these strains. A possible molecular player involved in hub tropism could be encoded by the gene region known as ‘octomom’. This region was found to be amplified several times in *w*MelPop, and contains genes predicted to be involved in DNA replication. It has been proposed to be responsible for the *w*MelPop over-replication phenotype [25], although there are conflicting reports [49]. This could explain the highest titers present in *w*MelPop-infected hubs. However, there are other unknown factors contributing to the range of hub tropism phenotypes observed in the other *w*MelCS-like and *w*Mel-like strains, since they have only once copy of the octomom region. The *w*Mel variants are defined by several polymorphic genetic markers [25], [44], [45], [49]. There are 108 single nucleotide polymorphisms (SNPs), a tandem duplication, and seven insertion-deletion polymorphisms between the *w*Mel and *w*MelCS-like (*w*MelPop) strains [25]. Further characterization of niche tropism of different strains in the same host genetic background, together with additional sequencing of diverse strains, will allow the correlation of *Wolbachia* genomic features with patterns of niche tropism. Future identification of *Wolbachia* proteins modulating the different levels of hub tropism will provide insights into the evolutionary mechanism driving this rapid divergence in males and the robust sexual dimorphism of stem cell niche targeting.

Here we presented tropism differences in *Wolbachia* strains well characterized at the genomic level in a *Drosophila* species with a large repertoire of transgenic and genetic tools. These findings provide the foundation to dissect the molecular mechanisms involved in *Wolbachia* hub tropism. Furthermore, the differences in stem cell niche tropism between males and females may reveal sex specific differences in the biology of stem cell niche being recognized by *Wolbachia*. Identification of the *Wolbachia* factors involved in tissue tropism is fundamental in understanding how bacteria spread and infect their hosts in nature and will provide additional tools towards vector and disease control.

## Materials and Methods

### Fly stocks used for analysis

Fly stocks used in this analysis and their sources are listed in S1 Table. *Drosophila* species naturally infected with *Wolbachia* comprising the *melanogaster* subgroup were selected, along with two additional species outside the *melanogaster* subgroup: *D. tropicalis* and *D. ananassae*, belonging to the *willistoni* and *ananassae* subgroups, respectively. Introgression crosses for hybrid analysis experiments were performed as previously described [14]. *D. melanogaster* flies infected with the several *w*Mel *Wolbachia* variants were introduced into the same genetic background as described elsewhere [25].

### Fly husbandry

Flies were raised at room temperature and fed a typical molasses, yeast, cornmeal, agar food, with the exception of *D. sechellia* flies which were supplemented with reconstituted Noni Fruit (Hawaiian Health Ohana, LLC) [50].

### Immunohistochemistry

For consistency and proper comparison to previous analysis of niche tropism in the female, males in this study were aged to seven days at room temperature (with the exception of the *D. simulans* hybrids for Fig. 4, which were dissected upon eclosion, see Toomey et al, 2013 for details). At least 20 flies were dissected for each sample, and total N's of hubs analyzed are listed in the Supplemental tables for each experiment. Testis were fixed using a 4% paraformaldehyde solution and subjected to immunostaining as previously described [13]. The mouse anti-hsp60 (Sigma, 1∶100) antibody was used to visualize *Wolbachia*. Hub markers were either rat anti-α-catenin (DSHB, DCAT1, 1∶40) or rat anti-DE-Cadherin (DSHB, DCAD2, 1∶20). Nuclei were counterstained with Hoechst (1 µg/ml, Molecular Probes).

### Image analysis of *Wolbachia* niche tropism

Images of the hub were acquired using a FV1000 confocal microscope. *Wolbachia* signal intensity in the hub and surrounding area were measured in Z-stacks of images using MatLab software for image quantification. Manual masks were drawn around the hub structure as well as the surrounding soma and germline using only the hub marker and DNA. *Wolbachia* density was measured within each mask and *Wolbachia* infection of the hub was considered tropism if the density relative to the surrounding soma and germline was at least 1.5-fold increased. A 1.5-fold threshold for tropism was previously determined to best represent what visually appears to be a higher density of *Wolbachia* in the niche versus the surrounding tissue [14]. Raw data showing density ratios is provided in S1 Dataset.

### Phylogenetic analyses

We utilized a computer simulation model of randomized character distributions to compare with the distribution of niche tropism pattern on each of the phylogenies to quantify the correlation of niche tropism pattern to the *Wolbachia* and *Drosophila* phylogenies (S1 and S3 Figs.) [51]. We used tree length as a measurement for goodness of fit for the distribution of a character, such as the tropism pattern, as aligned with the phylogeny. Tree length is defined as the total number of steps required to map a data set onto a phylogenetic tree.

### Statistical analysis of data

To determine the three significant groups for tropism in Fig. 1, a two-sample test for proportions was used on frequency data (Fig. 1K) and T-tests were used for density data (Fig. 1L). A Bonferroni correction was applied to account for multiple comparisons.

To determine the significance of host genetic background versus *Wolbachia* strain (Fig. 4) on the frequency of hub tropism a logistical regression was performed on frequency data as previously described (Fig. 4B) [14]. When “zero” frequencies are present, logistic regression analysis was replaced by a Fisher Exact Test (Fig. 3B). For density data, a linear regression was performed (Fig. 3C).

To determine if the frequencies of targeting between *Wolbachia* strains were significantly different (Fig. 5B), a two-sample test for proportions was used. If there were more than two strains being compared a Chi-square test was performed. To determine if the differences in densities were significant, pair-wise t-tests were performed (Fig. 5C).

## Supporting Information

S1 Fig ***Wolbachia*** **antibody staining controls.** (A) Antibody staining of a *Wolbachia* uninfected (W-) control. Hub marker in red, DNA in blue, Hsp60 staining of *Wolbachia* in green. Very little background staining occurs in a W- control. (B) In situ hybridization for *Wolbachia*. DNA in blue, a DNA probe against the *Wolbachia* 16S-rRNA is in green. (B′) Gray scale inset of *Wolbachia* channel in the hub. (B″) Gray scale inset of DNA in the hub. (C) Hsp60 antibody staining of *Wolbachia* infected testis. (C′) Gray scale inset of the *Wolbachia* channel only. (C″) Gray scale inset of DNA channel only. The inset shows haze of DNA stain for *Wolbachia* in the hub, along with brighter spots of A/T rich regions of host nuclear DNA (usually heterochromatic regions). *Wolbachia* present the same pattern of hub localization in both antibody staining and FISH (compare insets B′ and C′).
(TIF)

S2 Fig **Random fit distribution of niche tropism on the** ***Wolbachia*** **phylogeny.** (A) Hub tropism phenotype traced and character fit to the phylogeny. *Wolbachia* phylogeny adapted from [52]. Hub tropism traced onto the *Wolbachia* phylogeny requires 6 steps. (B) A set of 1000 random characters was computer simulated to assess the probability of the hub tropism character fit to the phylogeny due to chance. The probability of a fit as good, or better than the true character calculated for this phylogeny is a 100%. Simulations performed with MacClade Software [53], see Methods and Materials.
(TIF)

S3 Fig ***Wolbachia*** **tropism to the hub does not correlate with either the** ***Drosophila*** **or** ***Wolbachia*** **phylogenies.** Different patterns of niche targeting are correlated with *Drosophila* (left) and *Wolbachia* (right) phylogenies (phylogenies adapted from [52], [54]) (MYA =  million years ago). Green, blue, and red lines indicate high, moderate, and low frequency of hub tropism respectively. *wDin is a male killing strain of *Wolbachia*.
(TIF)

S4 Fig **Random fit distribution of niche tropism on** ***Drosophila*** **phylogenies.** (A) Hub tropism phenotype traced to the *Drosophila* phylogeny (adapted from [54]). Hub tropism traced onto the *Drosophila* phylogeny requires 5 steps. (B) A set of 1000 random characters was computer simulated to assess the probability of the hub tropism character fit to the phylogeny due to chance. The probability of a fit as good, or better than the true character calculated for this phylogeny is a 100%. Simulations performed with MacClade Software [53], see Methods and Materials.
(TIF)

S5 Fig **Hubs infected with** ***w*** **MelPop burst open.** (A–C) Representative images of hubs classified as normal high niche infection (HN, A), abnormal hub morphology suggestive of swelling, but not yet bursting (B), and bursting (C). *Wolbachia* is stained in green and the hub is in red. (A′–C′) insets of each image show the gray scale of the *Wolbachia* channel. (A″–C″) insets of each image show the gray scale of the hub marker. In the bursting hub (C″), it is evident that the hub cell membrane has been broken open. (D) Quantification of hub infection phenotype. Scale Bar is 5 µm.
(TIF)

S1 Table **Fly stocks utilized.** *Drosophila* species and their corresponding *Wolbachia* strains used for analysis are listed, along with their source and San Diego stock center number if applicable. **BOLD** indicates fly species with non-native *Wolbachia* strains introduced via hybrid crossing.
(PDF)

S2 Table **Frequencies and densities of** ***Wolbachia*** **hub tropism in diverse** ***Drosophila-Wolbachia*** **pairs.** Tropism for the hub was quantified using MatLab imaging software and confocal imaging (See materials and methods). For each individual fly, *Wolbachia* infection of the hub was qualified as “hub tropism” if the density was at least 1.5-fold higher in the hub than the surrounding tissue. Frequency shows the percent of flies that satisfied this criterion. The overall density of the species is shown.
(PDF)

S3 Table **Hub tropism does not correlate with GSCN tropism in the ovary.** The presence or absence of stem cell niche tropism in males was compared to previously determined tropism in the female GSCN *[14]. Frequencies from 0–9% are considered low/no tropism; 10–59% are considered moderate tropism; 60–100% are considered high tropism. Statistical correlation test shows no relationship between males and females (p = 0.773).
(PDF)

S4 Table **Frequency of** ***Wolbachia*** **targeting in** ***D. mauritiana, D. sechellia,*** **and the hybrid backcrossed F5 progeny.** Hybrid lines bolded.
(PDF)

S5 Table **Hub tropism does not correlate with cytoplasmic incompatibility.** Cytoplasmic incompatibility levels were obtained from each respective citation and correlated with frequencies of hub tropism. (Correlation test, p = 0.267).
(PDF)

S6 Table **Frequency of** ***Wolbachia*** **targeting in** ***D. simulans*** **and the hybrid backcrossed F5 progeny.** Hybrid lines bolded.
(PDF)

S7 Table **Frequencies and densities of** ***Wolbachia*** **hub tropism in** ***D. melanogaster*** **.** Tropism for the hub was quantified using MatLab software and confocal imaging. *Wolbachia* infection of the hub was considered tropism if the density was 1.5-fold higher in the hub than the surrounding tissue.
(PDF)

S1 Movie **High density of** ***w*** **MelPop infection in the hub.** 3-dimensional reconstruction of a confocal Z series of a *w*MelPop highly infected hub. Hub marker is shown in red and *Wolbachia* in green. Wolbachia clump below the hub is at the surface of the testis, in the muscle epithelia that covers the testis.
(AVI)

S2 Movie **Bursting** ***w*** **MelPop infected hub.** 3-dimensional reconstruction of a confocal Z series of a *w*MelPop infected hub with *Wolbachia* disrupting hub cells. Hub marker is shown in red and *Wolbachia* in green. On the top and bottom of the rotating hub, lysed hub cells release copious amounts of bacteria.
(AVI)

S1 Dataset **Raw data of** ***Wolbachia*** **density measurement for each hub analyzed.** Histogram in the first sheet shows the distribution of *Wolbachia* density ratios in the different *Wolbachia* strains analyzed. *Wolbachia* density was quantified for each hub imaged as described in material and methods. Each sheet in the spreadsheet corresponds to a *Drosophila* – *Wolbachia* pair. Values are arbitrary units that correspond to an intensity value for each pixel converted to grayscale. Values in columns B and C indicate the *Wolbachia* density. The values correspond to pixel intensity measured in the *Wolbachia* channel (green) divided by the area in each Z plane, either in the hub or surrounding tissue (germline and soma). At least 3 different Z planes were used for each hub imaged. Column D shows the *Wolbachia* density ratio of hub to surrounding cells for each individual hub with average and standard deviation at the bottom. Columns E – I characterize the density range category utilized in the histogram.
(XLSX)

## References

[1] Chapman T, Arnqvist G, Bangham J, Rowe L (2003) Sexual conflict. Trends in Ecology & Evolution 18: 41–47.

[2] Avila FW, Sirot LK, LaFlamme BA, Rubinstein CD, Wolfner MF (2011) Insect seminal fluid proteins: identification and function. Annu Rev Entomol 56: 21–40. 20868282 10.1146/annurev-ento-120709-144823 PMC3925971

[3] Baldo L, Ayoub NA, Hayashi CY, Russell JA, Stahlhut JK, et al (2008) Insight into the routes of Wolbachia invasion: high levels of horizontal transfer in the spider genus Agelenopsis revealed by Wolbachia strain and mitochondrial DNA diversity. Molecular ecology 17: 557–569. 18179432 10.1111/j.1365-294X.2007.03608.x

[4] Schilthuizen M, Stouthamer R (1997) Horizontal transmission of parthenogenesis-inducing microbes in Trichogramma wasps. Proc R Soc Lond B Biol Sci 264: 361–366. 10.1098/rspb.1997.0052 PMC1688260 9107051

[5] Werren JH (2011) Selfish genetic elements, genetic conflict, and evolutionary innovation. Proceedings of the National Academy of Sciences of the United States of America 108 Suppl 2: 10863–10870. 21690392 10.1073/pnas.1102343108 PMC3131821

[6] Werren JH, Baldo L, Clark ME (2008) Wolbachia: master manipulators of invertebrate biology. Nat Rev Microbiol 6: 741–751. 18794912 10.1038/nrmicro1969

[7] Ferree PM, Frydman, H M, Li JM, Cao J, Wieschaus E, Sullivan W (2005) Wolbachia Utilizes Host Microtubules and Dynein for Anterior Localization in the Drosophila Oocyte. PLoS Pathog 1: 111–124 (e114).. 10.1371/journal.ppat.0010014 PMC1253842 16228015

[8] Hadfield SJ, Axton JM (1999) Germ cells colonized by endosymbiotic bacteria. Nature 402: 482. 10591206 10.1038/45002

[9] Serbus LR, Sullivan W (2007) A cellular basis for Wolbachia recruitment to the host germline. PLoS Pathog 3: e190. 18085821 10.1371/journal.ppat.0030190 PMC2134955

[10] Veneti Z, Clark ME, Karr TL, Savakis C, Bourtzis K (2004) Heads or tails: host-parasite interactions in the Drosophila-Wolbachia system. Appl Environ Microbiol 70: 5366–5372. 15345422 10.1128/AEM.70.9.5366-5372.2004 PMC520876

[11] Boyle L, Oneill SL, Robertson HM, Karr TL (1993) Interspecific and Intraspecific Horizontal Transfer of Wolbachia in Drosophila. Science 260: 1796–1799. 8511587 10.1126/science.8511587

[12] Vavre F, Fleury F, Lepetit D, Fouillet P, Bouletreau M (1999) Phylogenetic evidence for horizontal transmission of Wolbachia in host- parasitoid associations. Mol Biol Evol 16: 1711–1723. 10605113 10.1093/oxfordjournals.molbev.a026084

[13] Frydman HM, Li JM, Robson DN, Wieschaus E (2006) Somatic stem cell niche tropism in Wolbachia. Nature 441: 509–512. 16724067 10.1038/nature04756

[14] Toomey ME, Panaram K, Fast EM, Beatty C, Frydman HM (2013) Evolutionarily conserved Wolbachia-encoded factors control pattern of stem-cell niche tropism in Drosophila ovaries and favor infection. Proc Natl Acad Sci U S A 110: 10788–10793. 23744038 10.1073/pnas.1301524110 PMC3696799

[15] Hosokawa T, Koga R, Kikuchi Y, Meng XY, Fukatsu T (2010) Wolbachia as a bacteriocyte-associated nutritional mutualist. Proc Natl Acad Sci U S A 107: 769–774. 20080750 10.1073/pnas.0911476107 PMC2818902

[16] Sacchi L, Genchi M, Clementi E, Negri I, Alma A, et al (2010) Bacteriocyte-like cells harbour Wolbachia in the ovary of Drosophila melanogaster (Insecta, Diptera) and Zyginidia pullula (Insecta, Hemiptera). Tissue Cell 42: 328–333. 20817243 10.1016/j.tice.2010.07.009

[17] Fast EM, Toomey ME, Panaram K, Desjardins D, Kolaczyk ED, et al (2011) Wolbachia enhance Drosophila stem cell proliferation and target the germline stem cell niche. Science 334: 990–992. 22021671 10.1126/science.1209609 PMC4030408

[18] Hardy RW, Tokuyasu KT, Lindsley DL, Garavito M (1979) The germinal proliferation center in the testis of Drosophila melanogaster. J Ultrastruct Res 69: 180–190. 114676 10.1016/s0022-5320(79)90108-4

[19] Charlat S, Nirgianaki A, Bourtzis K, Mercot H (2002) Evolution of Wolbachia-induced cytoplasmic incompatibility in Drosophila simulans and D. sechellia. Evolution Int J Org Evolution 56: 1735–1742. 10.1111/j.0014-3820.2002.tb00187.x 12389718

[20] Zabalou S, Charlat S, Nirgianaki A, Lachaise D, Mercot H, et al (2004) Natural Wolbachia infections in the Drosophila yakuba species complex do not induce cytoplasmic incompatibility but fully rescue the wRi modification. Genetics 167: 827–834. 15238531 10.1534/genetics.103.015990 PMC1470911

[21] van Meer MMM, Witteveldt J, Stouthamer R (1999) Phylogeny of the arthropod endosymbiont Wolbachia based on the wsp gene. Insect Molecular Biology 8: 399–408. 10469257 10.1046/j.1365-2583.1999.83129.x

[22] Veneti Z, Clark ME, Zabalou S, Karr TL, Savakis C, et al (2003) Cytoplasmic incompatibility and sperm cyst infection in different Drosophila-Wolbachia associations. Genetics 164: 545–552. 12807775 10.1093/genetics/164.2.545 PMC1462605

[23] Bourtzis K, Nirgianaki A, Markakis G, Savakis C (1996) Wolbachia infection and cytoplasmic incompatibility in Drosophila species. Genetics 144: 1063–1073. 8913750 10.1093/genetics/144.3.1063 PMC1207602

[24] Richardson MF, Weinert LA, Welch JJ, Linheiro RS, Magwire MM, et al (2012) Population genomics of the Wolbachia endosymbiont in Drosophila melanogaster. PLoS Genet 8: e1003129. 23284297 10.1371/journal.pgen.1003129 PMC3527207

[25] Chrostek E, Marialva MS, Esteves SS, Weinert LA, Martinez J, et al (2013) Wolbachia variants induce differential protection to viruses in Drosophila melanogaster: a phenotypic and phylogenomic analysis. PLoS Genet 9: e1003896. 24348259 10.1371/journal.pgen.1003896 PMC3861217

[26] Landmann F, Bain O, Martin C, Uni S, Taylor MJ, et al (2012) Both asymmetric mitotic segregation and cell-to-cell invasion are required for stable germline transmission of Wolbachia in filarial nematodes. Biol Open 1: 536–547. 23213446 10.1242/bio.2012737 PMC3509449

[27] Decotto E, Spradling AC (2005) The Drosophila ovarian and testis stem cell niches: similar somatic stem cells and signals. Dev Cell 9: 501–510. 16198292 10.1016/j.devcel.2005.08.012

[28] Gilboa L, Lehmann R (2004) How different is Venus from Mars? The genetics of germ-line stem cells in Drosophila females and males. Development 131: 4895–4905. 15459096 10.1242/dev.01373

[29] Werren JH (1997) Biology of Wolbachia. Annu Rev Entomol 42: 587–609. 15012323 10.1146/annurev.ento.42.1.587

[30] Tram U, Sullivan W (2002) Role of delayed nuclear envelope breakdown and mitosis in Wolbachia-induced cytoplasmic incompatibility. Science 296: 1124–1126. 12004132 10.1126/science.1070536

[31] Pinto SB, Stainton K, Harris S, Kambris Z, Sutton ER, et al (2013) Transcriptional regulation of Culex pipiens mosquitoes by Wolbachia influences cytoplasmic incompatibility. PLoS Pathog 9: e1003647. 24204251 10.1371/journal.ppat.1003647 PMC3814344

[32] O'Neill SL, Karr TL (1990) Bidirectional incompatibility between conspecific populations of Drosophila simulans. Nature 348: 178–180. 2234083 10.1038/348178a0

[33] Breeuwer JA, Werren JH (1990) Microorganisms associated with chromosome destruction and reproductive isolation between two insect species. Nature 346: 558–560. 2377229 10.1038/346558a0

[34] Brennan LJ, Haukedal JA, Earle JC, Keddie B, Harris HL (2012) Disruption of redox homeostasis leads to oxidative DNA damage in spermatocytes of Wolbachia-infected Drosophila simulans. Insect Mol Biol 21: 510–520. 22831171 10.1111/j.1365-2583.2012.01155.x

[35] Riparbelli MG, Giordano R, Callaini G (2007) Effects of Wolbachia on sperm maturation and architecture in Drosophila simulans Riverside. Mech Dev 124: 699–714. 17693061 10.1016/j.mod.2007.07.001

[36] Cherry CM, Matunis EL (2010) Epigenetic regulation of stem cell maintenance in the Drosophila testis via the nucleosome-remodeling factor NURF. Cell Stem Cell 6: 557–567. 20569693 10.1016/j.stem.2010.04.018 PMC2897240

[37] Haerty W, Jagadeeshan S, Kulathinal RJ, Wong A, Ravi Ram K, et al (2007) Evolution in the fast lane: rapidly evolving sex-related genes in Drosophila. Genetics 177: 1321–1335. 18039869 10.1534/genetics.107.078865 PMC2147986

[38] Bauer DuMont VL, Flores HA, Wright MH, Aquadro CF (2007) Recurrent positive selection at bgcn, a key determinant of germ line differentiation, does not appear to be driven by simple coevolution with its partner protein bam. Mol Biol Evol 24: 182–191. 17056645 10.1093/molbev/msl141

[39] Baldo L, Bordenstein S, Wernegreen JJ, Werren JH (2006) Widespread recombination throughout Wolbachia genomes. Mol Biol Evol 23: 437–449. 16267140 10.1093/molbev/msj049

[40] Klasson L, Westberg J, Sapountzis P, Naslund K, Lutnaes Y, et al (2009) The mosaic genome structure of the Wolbachia wRi strain infecting Drosophila simulans. Proceedings of the National Academy of Sciences of the United States of America 106: 5725–5730. 19307581 10.1073/pnas.0810753106 PMC2659715

[41] Baldo L, Desjardins CA, Russell JA, Stahlhut JK, Werren JH (2010) Accelerated microevolution in an outer membrane protein (OMP) of the intracellular bacteria Wolbachia. BMC Evol Biol 10: 48. 20163713 10.1186/1471-2148-10-48 PMC2843615

[42] Siozios S, Ioannidis P, Klasson L, Andersson SG, Braig HR, et al (2013) The diversity and evolution of Wolbachia ankyrin repeat domain genes. PLoS One 8: e55390. 23390535 10.1371/journal.pone.0055390 PMC3563639

[43] Choi JY, Aquadro CF (2014) The coevolutionary period of Wolbachia pipientis infecting Drosophila ananassae and its impact on the evolution of the host germline stem cell regulating genes. Mol Biol Evol 31: 2457–2471. 24974378 10.1093/molbev/msu204 PMC4137719

[44] Riegler M, Sidhu M, Miller WJ, O'Neill SL (2005) Evidence for a global Wolbachia replacement in Drosophila melanogaster. Curr Biol 15: 1428–1433. 16085497 10.1016/j.cub.2005.06.069

[45] Ilinsky Y (2013) Coevolution of Drosophila melanogaster mtDNA and Wolbachia genotypes. PLoS One 8: e54373. 23349865 10.1371/journal.pone.0054373 PMC3547870

[46] Jiggins FM, Hurst GD, Yang Z (2002) Host-symbiont conflicts: positive selection on an outer membrane protein of parasitic but not mutualistic Rickettsiaceae. Mol Biol Evol 19: 1341–1349. 12140246 10.1093/oxfordjournals.molbev.a004195

[47] Brownlie JC, Adamski M, Slatko B, McGraw EA (2007) Diversifying selection and host adaptation in two endosymbiont genomes. BMC Evol Biol 7: 68. 17470297 10.1186/1471-2148-7-68 PMC1868728

[48] Moran NA, McCutcheon JP, Nakabachi A (2008) Genomics and evolution of heritable bacterial symbionts. Annu Rev Genet 42: 165–190. 18983256 10.1146/annurev.genet.41.110306.130119

[49] Woolfit M, Iturbe-Ormaetxe I, Brownlie JC, Walker T, Riegler M, et al (2013) Genomic evolution of the pathogenic Wolbachia strain, wMelPop. Genome Biol Evol 5: 2189–2204. 24190075 10.1093/gbe/evt169 PMC3845649

[50] Amlou M, Moreteau B, David JR (1998) Genetic analysis of Drosophila sechellia specialization: oviposition behavior toward the major aliphatic acids of its host plant. Behavior genetics 28: 455–464. 9926614 10.1023/a:1021689312582

[51] Maddison WP, Maddison DR (2005) MacClade; Analysis of phylogeny and character evolution. 4.08a ed. Sunderland, Massachussettz: Sinauer Associates. Pp.

[52] Paraskevopoulos C, Bordenstein SR, Wernegreen JJ, Werren JH, Bourtzis K (2006) Toward a Wolbachia multilocus sequence typing system: discrimination of Wolbachia strains present in Drosophila species. Curr Microbiol 53: 388–395. 17036209 10.1007/s00284-006-0054-1

[53] Maddison WP, Maddison DR (2005) MacClade: Analysis of phylogeny and character evolution. 4.08a ed. Sunderland, Massachusetts: Sinauer Associates.

[54] Jeffs PS, Holmes EC, Ashburner M (1994) The molecular evolution of the alcohol dehydrogenase and alcohol dehydrogenase-related genes in the Drosophila melanogaster species subgroup. Mol Biol Evol 11: 287–304. 8170369 10.1093/oxfordjournals.molbev.a040110

