# Supplementary figures and images for: Extreme Divergence of Wolbachia Tropism for the Stem-Cell-Niche in the Drosophila Testis
Source: PLoS Pathog. 2014 Dec 18;10(12):e1004577. doi: 10.1371/journal.ppat.1004577 (PMC4270793; doi:10.1371/journal.ppat.1004577)

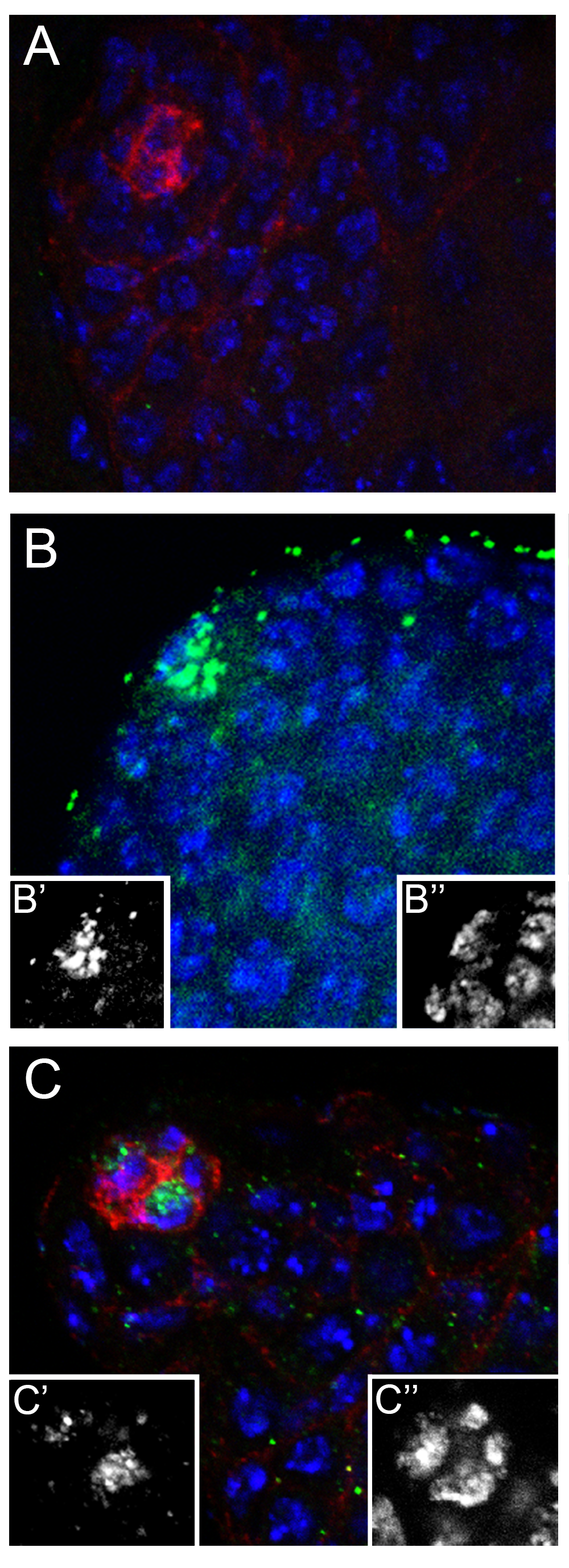

Supplement: S1 Fig — Wolbachia antibody staining controls. (A) Antibody staining of a Wolbachia uninfected (W-) control. Hub marker in red, DNA in blue, Hsp60 staining of Wolbachia in green. Very little background staining occurs in a W- control. (B) In situ hybridization for Wolbachia. DNA in blue, a DNA probe against the Wolbachia 16S-rRNA is in green. (B′) Gray scale inset of Wolbachia channel in the hub. (B″) Gray scale inset of DNA in the hub. (C) Hsp60 antibody staining of Wolbachia infected testis. (C′) Gray scale inset of the Wolbachia channel only. (C″) Gray scale inset of DNA channel only. The inset shows haze of DNA stain for Wolbachia in the hub, along with brighter spots of A/T rich regions of host nuclear DNA (usually heterochromatic regions). Wolbachia present the same pattern of hub localization in both antibody staining and FISH (compare insets B′ and C′). (TIF) [file ppat.1004577.s001.tif]

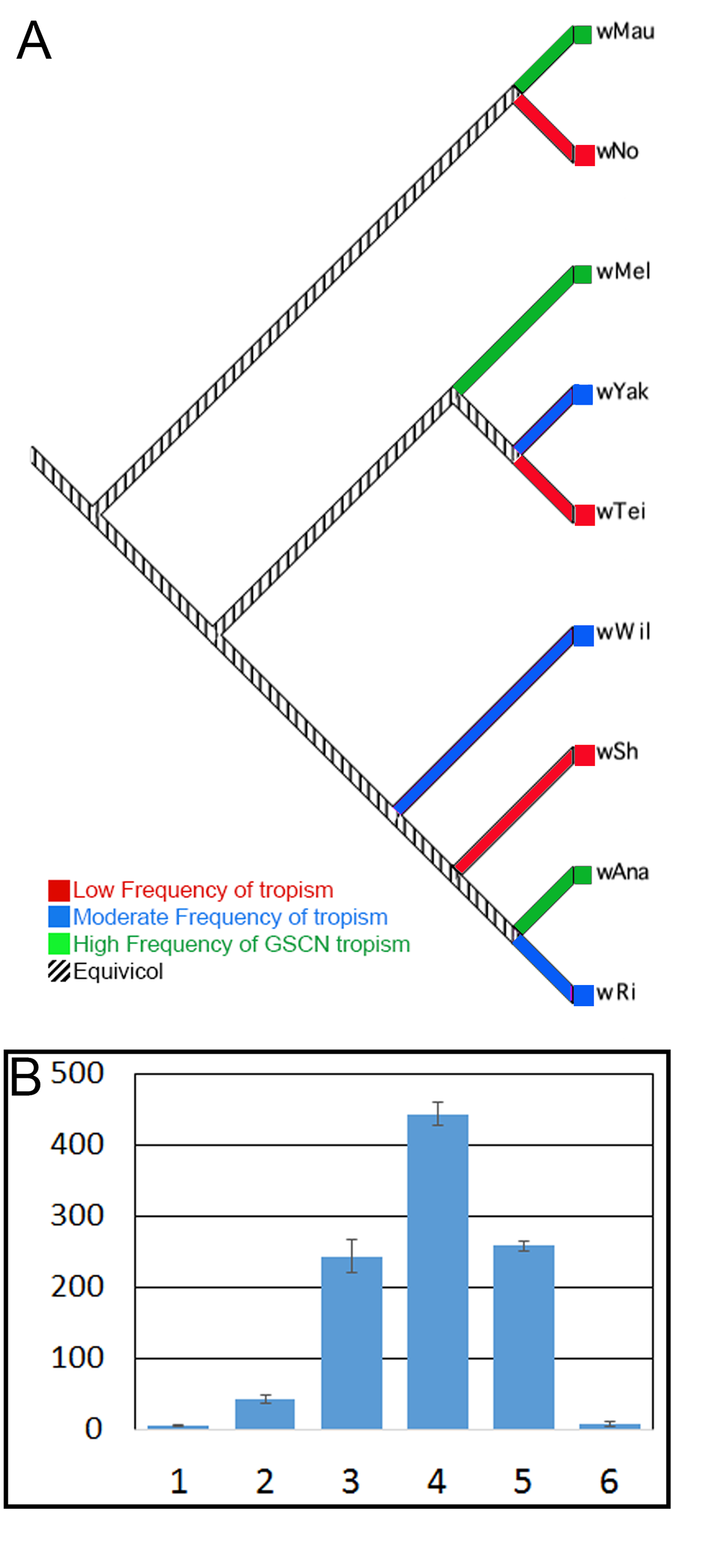

Supplement: S2 Fig — Random fit distribution of niche tropism on the Wolbachia phylogeny. (A) Hub tropism phenotype traced and character fit to the phylogeny. Wolbachia phylogeny adapted from [52]. Hub tropism traced onto the Wolbachia phylogeny requires 6 steps. (B) A set of 1000 random characters was computer simulated to assess the probability of the hub tropism character fit to the phylogeny due to chance. The probability of a fit as good, or better than the true character calculated for this phylogeny is a 100%. Simulations performed with MacClade Software [53], see Methods and Materials. (TIF) [file ppat.1004577.s002.tif]

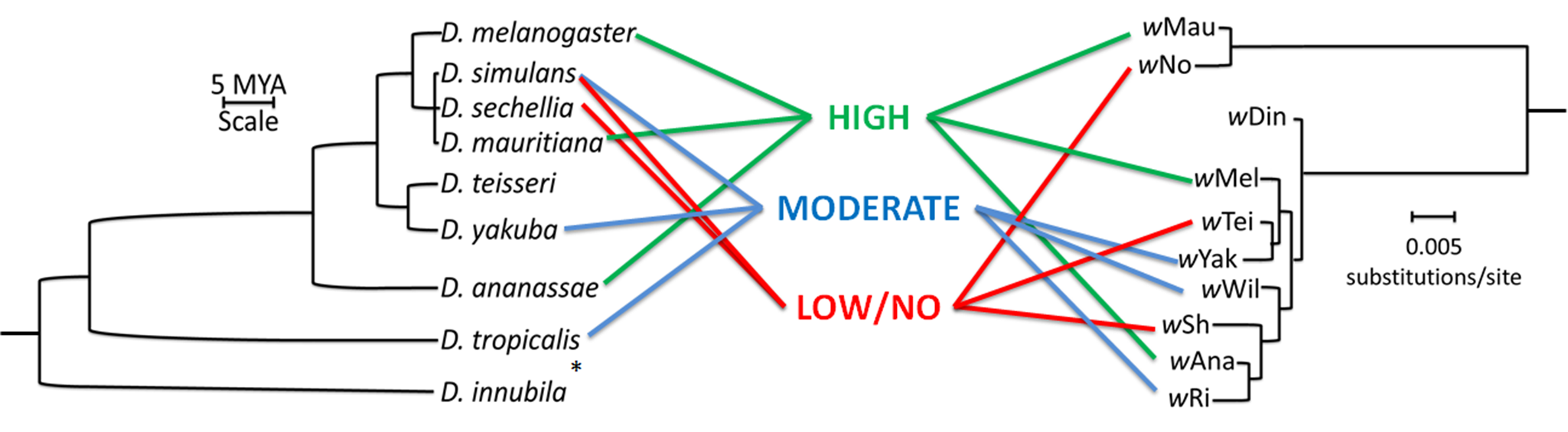

Supplement: S3 Fig — Wolbachia tropism to the hub does not correlate with either the Drosophila or Wolbachia phylogenies. Different patterns of niche targeting are correlated with Drosophila (left) and Wolbachia (right) phylogenies (phylogenies adapted from [52], [54]) (MYA = million years ago). Green, blue, and red lines indicate high, moderate, and low frequency of hub tropism respectively. *wDin is a male killing strain of Wolbachia. (TIF) [file ppat.1004577.s003.tif]

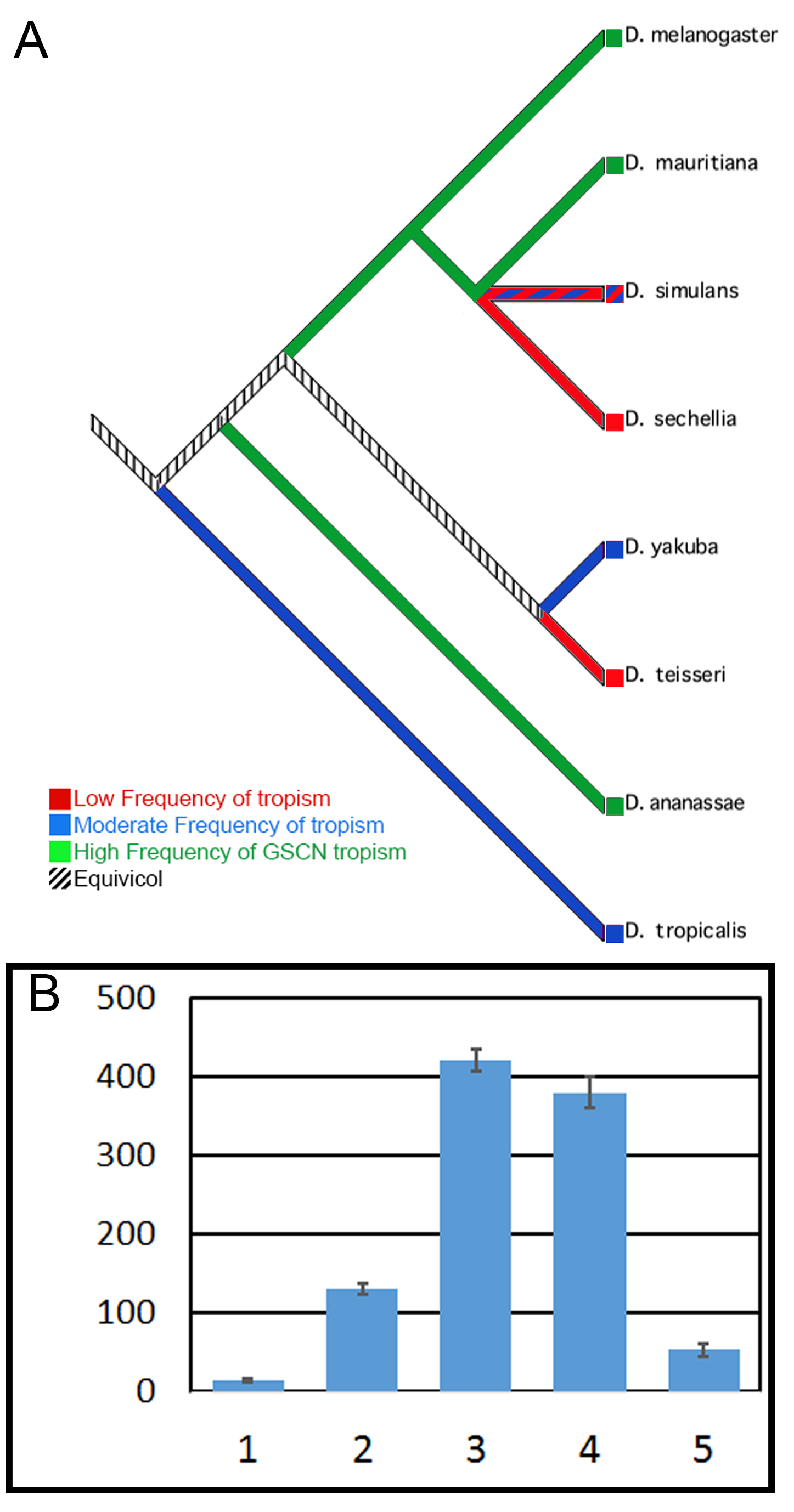

Supplement: S4 Fig — Random fit distribution of niche tropism on Drosophila phylogenies. (A) Hub tropism phenotype traced to the Drosophila phylogeny (adapted from [54]). Hub tropism traced onto the Drosophila phylogeny requires 5 steps. (B) A set of 1000 random characters was computer simulated to assess the probability of the hub tropism character fit to the phylogeny due to chance. The probability of a fit as good, or better than the true character calculated for this phylogeny is a 100%. Simulations performed with MacClade Software [53], see Methods and Materials. (TIF) [file ppat.1004577.s004.tif]

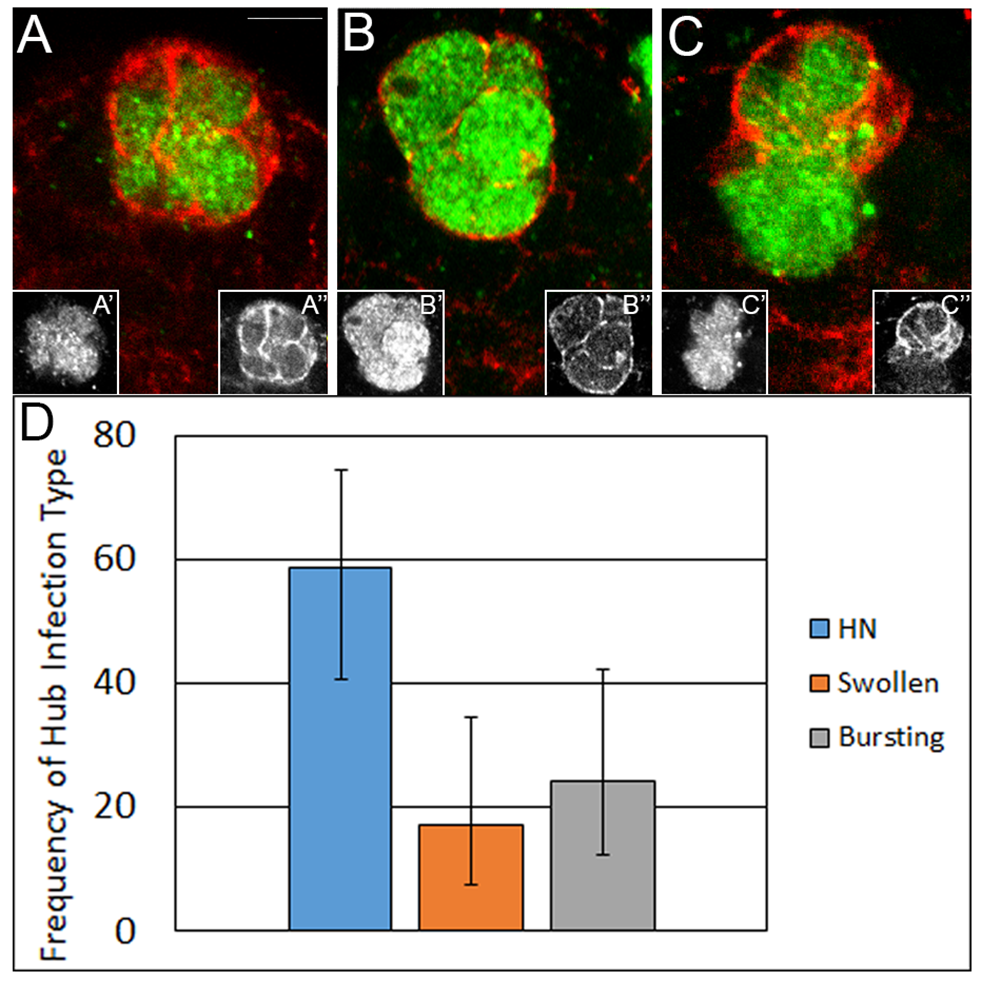

Supplement: S5 Fig — Hubs infected with w MelPop burst open. (A–C) Representative images of hubs classified as normal high niche infection (HN, A), abnormal hub morphology suggestive of swelling, but not yet bursting (B), and bursting (C). Wolbachia is stained in green and the hub is in red. (A′–C′) insets of each image show the gray scale of the Wolbachia channel. (A″–C″) insets of each image show the gray scale of the hub marker. In the bursting hub (C″), it is evident that the hub cell membrane has been broken open. (D) Quantification of hub infection phenotype. Scale Bar is 5 µm. (TIF) [file ppat.1004577.s005.tif]
