# Supplementary material for: Extreme Divergence of Wolbachia Tropism for the Stem-Cell-Niche in the Drosophila Testis
Source: PLoS Pathog. 2014 Dec 18;10(12):e1004577. doi: 10.1371/journal.ppat.1004577 (PMC4270793; doi:10.1371/journal.ppat.1004577)
Supplement: S1 Table — Fly stocks utilized. Drosophila species and their corresponding Wolbachia strains used for analysis are listed, along with their source and San Diego stock center number if applicable. BOLD indicates fly species with non-native Wolbachia strains introduced via hybrid crossing. (PDF) [file ppat.1004577.s006.pdf]

Drosophila Species	*Wolbachia* Strain	Source	Stock Center #/Reference
D. melanogaster	*w*Mel	Frydman Lab	–
D. simulans	*w*No	San Diego Stock Center	14021-0251.198
D. simulans	*w*Ri	San Diego Stock Center	14021-0251.169
D. sechellia	*w*Sh	San Diego Stock Center	14021-0248.08
D. mauritiana	*w*Mau	San Diego Stock Center	14021-0241.01
D. teissieri	*w*Tei	San Diego Stock Center	14021-0257.00
D. yakuba	*w*Yak	Virginie Orgogozo	–
D. tropicalis	*w*Wil	San Diego Stock Center	14030-0801.01
D. ananassae	*w*Ana	Jack Werren/Michael Clark	–
D. mauritiana	***w*Sh**	Frydman Lab	–
D. sechellia	***w*Mau**	Frydman Lab	–
D. simulans	***w*Ri**	Frydman Lab	–
D. simulans	***w*No**	Frydman Lab	–
D. melanogaster	*w*Mel	Luis Teixeira	Chrostek *et al.*, 2013
D. melanogaster	*w*Mel2*	Luis Teixeira	Chrostek *et al.*, 2013
D. melanogaster	*w*Mel3	Luis Teixeira	Chrostek *et al.*, 2013
D. melanogaster	*w*MelCS*	Luis Teixeira	Chrostek *et al.*, 2013
D. melanogaster	*w*MelCS2*	Luis Teixeira	Chrostek *et al.*, 2013
D. melanogaster	*w*MelPop	Luis Teixeira	Chrostek *et al.*, 2013
