# Supplementary material for: Extreme Divergence of Wolbachia Tropism for the Stem-Cell-Niche in the Drosophila Testis
Source: PLoS Pathog. 2014 Dec 18;10(12):e1004577. doi: 10.1371/journal.ppat.1004577 (PMC4270793; doi:10.1371/journal.ppat.1004577)
Supplement: S2 Table — Frequencies and densities of Wolbachia hub tropism in diverse Drosophila-Wolbachia pairs. Tropism for the hub was quantified using MatLab imaging software and confocal imaging (See materials and methods). For each individual fly, Wolbachia infection of the hub was qualified as “hub tropism” if the density was at least 1.5-fold higher in the hub than the surrounding tissue. Frequency shows the percent of flies that satisfied this criterion. The overall density of the species is shown. (PDF) [file ppat.1004577.s007.pdf]

| Species | *Wolbachia* strain | N | Frequency | Density |
|---|---|---|---|---|
| *D. sechellia* | wSh | 44 | 0.00% | 0.491 |
| *D. simulans* | wNo | 30 | 0.00% | 0.616 |
| *D. teissieri* | wTei | 32 | 3.13% | 0.336 |
| *D. simulans* | wRi | 29 | 17.24% | 0.867 |
| *D. tropicalis* | wWil | 28 | 17.86% | 0.861 |
| *D. yakuba* | wYak | 32 | 28.13% | 0.881 |
| *D. mauritiana* | wMau | 27 | 66.67% | 4.378 |
| *D. melanogaster* | wMel | 35 | 71.43% | 4.871 |
| *D. ananassae* | wAna | 30 | 86.67% | 4.898 |
