# Supplementary material for: Extreme Divergence of Wolbachia Tropism for the Stem-Cell-Niche in the Drosophila Testis
Source: PLoS Pathog. 2014 Dec 18;10(12):e1004577. doi: 10.1371/journal.ppat.1004577 (PMC4270793; doi:10.1371/journal.ppat.1004577)
Supplement: S3 Table — Hub tropism does not correlate with GSCN tropism in the ovary. The presence or absence of stem cell niche tropism in males was compared to previously determined tropism in the female GSCN *[14]. Frequencies from 0–9% are considered low/no tropism; 10–59% are considered moderate tropism; 60–100% are considered high tropism. Statistical correlation test shows no relationship between males and females (p = 0.773). (PDF) [file ppat.1004577.s008.pdf]

Drosophila Species	*Wolbachia* Strain	Frequency of Hub Tropism	Frequency of GSCN Ovary Tropism*
D. sechellia	wSh	0	0.83
D. simulans	wNo	0	99.3
D. teissierri	wTei	3.13	3.6
D. simulans	wRi	17.24	53.7
D. tropicalis	wWil	17.86	32.7
D. yakuba	wYak	28.13	0.91
D. mauritiana	wMau	66.67	96
D. melanogaster	wMel	71.43	1
D. ananassae	wAna	83.61	47.7
