# Supplementary material for: Extreme Divergence of Wolbachia Tropism for the Stem-Cell-Niche in the Drosophila Testis
Source: PLoS Pathog. 2014 Dec 18;10(12):e1004577. doi: 10.1371/journal.ppat.1004577 (PMC4270793; doi:10.1371/journal.ppat.1004577)
Supplement: S4 Table — Frequency of Wolbachia targeting in D. mauritiana, D. sechellia, and the hybrid backcrossed F5 progeny. Hybrid lines bolded. (PDF) [file ppat.1004577.s009.pdf]

Wolbachia Strain	Frequency of Hub Tropism (%)	Levels of CI
wSh	0	high (Charlat et al, 2002)
wNo	0	mod (Charlat et al, 2002; Zabalou *et al* ,2004)
wTei	2.3	low (Zabalou *et al* ,2004)
wRi	17.24	high (Van Meer *et al,* 1999)
wWil	17.86	unknown
wYak	29.73	low (Zabalou *et al* ,2004)
wMau	65.52	low (Veneti *et al,* 2003)
wMel	70.83	low (Van Meer *et al,* 1999)
wAna	83.61	mod (Bourtzis *et al*, 1996)
wMelPop	94.52	low (Van Meer *et al,* 1999; Veneti *et al,* 2003)
