# Supplementary material for: Extreme Divergence of Wolbachia Tropism for the Stem-Cell-Niche in the Drosophila Testis
Source: PLoS Pathog. 2014 Dec 18;10(12):e1004577. doi: 10.1371/journal.ppat.1004577 (PMC4270793; doi:10.1371/journal.ppat.1004577)
Supplement: S5 Table — Hub tropism does not correlate with cytoplasmic incompatibility. Cytoplasmic incompatibility levels were obtained from each respective citation and correlated with frequencies of hub tropism. (Correlation test, p = 0.267). (PDF) [file ppat.1004577.s010.pdf]

Species	*Wolbachia* strain	N	%	Density
D. mauritiana	wMau	29	65.52%	4.378
D.sechellia	**wMau**	42	16.67%	2.649
D.sechellia	wSh	51	0.00%	0.491
D. mauritiana	**wSh**	27	0.00%	0.8573
