# Supplementary material for: Extreme Divergence of Wolbachia Tropism for the Stem-Cell-Niche in the Drosophila Testis
Source: PLoS Pathog. 2014 Dec 18;10(12):e1004577. doi: 10.1371/journal.ppat.1004577 (PMC4270793; doi:10.1371/journal.ppat.1004577)
Supplement: S6 Table — Frequency of Wolbachia targeting in D. simulans and the hybrid backcrossed F5 progeny. Hybrid lines bolded. (PDF) [file ppat.1004577.s011.pdf]

Species	*Wolbachia* strain	N	%
D. simulans 169	wRi	43	32.56%
***D. simulans* 198**	**wRi**	37	43.24%
D. simulans 198	wNo	43	2.33%
***D. simulans* 169**	**wNo**	34	14.71%
