# Supplementary material for: Extreme Divergence of Wolbachia Tropism for the Stem-Cell-Niche in the Drosophila Testis
Source: PLoS Pathog. 2014 Dec 18;10(12):e1004577. doi: 10.1371/journal.ppat.1004577 (PMC4270793; doi:10.1371/journal.ppat.1004577)
Supplement: S7 Table — Frequencies and densities of Wolbachia hub tropism in D. melanogaster . Tropism for the hub was quantified using MatLab software and confocal imaging. Wolbachia infection of the hub was considered tropism if the density was 1.5-fold higher in the hub than the surrounding tissue. (PDF) [file ppat.1004577.s012.pdf]

Wolbachia Strain	N	HN	Frequency	Density
*w*Mel	30	13	43.33	1.46
*w*Mel2	30	8	26.67	1.37
*w*Mel3	30	14	46.67	1.54
*w*MelCS2	30	23	76.67	11.67
*w*MelCS	30	27	90.00	7.81
*w*MelPop	30	30	100.00	47.99
